# Ex vivo study correlating the stiffness of the ovine patellar tendon to age and weight

**DOI:** 10.1080/23335432.2022.2108899

**Published:** 2022-08-05

**Authors:** Françoise Kayser, Edoardo Bori, Sophie Fourny, Fanny Hontoir, Peter Clegg, Alexandra Dugdale, Jean-Michel Vandeweerd, Bernardo Innocenti

**Affiliations:** a Department of Medical Imaging, CHU UCL NAMUR (Centre Hospitalier Universitaire-Université Catholique de Louvain-NAMUR) site Godinne, Yvoir, Belgium; b BEAMS Department (Bio-Electro and Mechanical System), ULB (Université Libre de Bruxelles)-Ecole Polytechnique de Bruxelles, Bruxelles, Belgium; c Department of Veterinary Medicine, University of Namur, Namur, Belgium; d NaRILiS (Namur Research Institute for Life Sciences)-IRVU (Integrated Veterinary Research Unit), Department of Veterinary Medicine, University of Namur, Namur, Belgium; e Faculty of Health and Life Sciences, Department of Musculoskeletal Biology, University of Liverpool, Neston, UK; f Units E & F, Telford Court, Dunkirk Trading Estate, Gates Lane, Chester Gates Veterinary Specialists CVS (UK) Ltd, Chester, UK; g BEAMS Department (Bio-Electro and Mechanical System), ULB (Université Libre de Bruxelles), Bruxelles, Belgium

**Keywords:** Age, ovine, patellar tendon, stiffness, weight

## Abstract

Tendons play a crucial role in the musculoskeletal system. In humans, tendon injuries, especially chronic tendinopathy, are very common and the patellar tendon is a frequent location for tendinopathy or injuries. The biomechanical characteristics of the patellar tendon, such as elasticity and stiffness, are of paramount importance and constitute major outcome measures in research studies. We aimed to assess whether the stiffness of the healthy ovine patellar tendon changes with age and weight in a population of normal animals. Sixty-eight ‘patella-patellar tendon-tibial tuberosity’ units from thirty-four Ile-de-France ewes of body mass 65 to 95 kg, euthanized for reasons other than musculoskeletal diseases, underwent a tensile test providing a measure of the tendon stiffness. Animals were sorted into three categories of age (1–2 yo, 3–5 yo, 6–10 yo). We found a positive but not significant correlation between age category and stiffness (r = 0.22, p = 0.27). There was a significantly positive correlation between weight and stiffness (r = 0.39, p = 0.04). In conclusion, the study characterized biomechanical properties of healthy tendons, provided useful reference values, and established the basis for future biomechanical tests on healing tendons in sheep. The most appropriate sheep population for those future studies would be non-overweight young adults presenting with no lameness.

## Introduction

Tendons play a crucial role in the musculoskeletal system. In humans, tendon injuries, especially chronic tendinopathy, are very common in occupational and athletic settings, and in the elderly population. They may be associated with significant morbidity and abnormal joint movement, and are an important cause of musculoskeletal disabilities or pain.

In particular, patellar tendon injuries occur either naturally as chronic tendinosis, partial/full-thickness tendon tears, acute avulsion fractures (Peace et al. 2006), or as artificially-created lesions when the patellar tendon is used as a donor site for tendon autograft in anterior cruciate ligament (Benner et al. 2012) and medial patellofemoral ligament reconstruction (Witoński et al. 2013).

Because of the restricted self-restoring capacity of tendon, spontaneous tendon healing often results in fibrous adhesions and inferior fibrotic scar tissue which is both mechanically – and functionally inferior (Sharma and Maffulli 2005). The tendon is also prone to re-injury.

Functional recovery of tendon injuries is challenging. Currently, treatment solutions are disappointing and healed tendons do not recuperate the biomechanical properties of intact tendons. There is, therefore, a need for an improved therapeutic approach (Gaida and Cook 2011; Charousset et al. 2014; Nanos and Malanga 2015; Ode and al. 2016).

In orthopaedic research, to develop new technologies, preclinical studies using animal models are needed (Cook and al. 2014). The need to mimic the dimensions and loading experienced by an adult human joint requires that large animal models of disease are employed. The use of the sheep as an animal model enables larger sampling populations, lameness assessment and scoring, and the use of non-invasive imaging in longitudinal studies. One of the advantages of the sheep over other large animal models is the human-like size of joints such as the knee (Little and Smith 2008). The anatomy of the ovine knee (stifle) has been well-characterized (Proffen and al. 2012). Ultrasonographic, CT and MRI anatomy of the stifle have been described (Vandeweerd et al. 2012; Vandeweerd et al. 2013; Kayser et al. 2019).

Since the patellar tendon covers a key-role in extension of the knee joint, the biomechanical characteristics of the patellar tendon, such as elasticity and stiffness, are of paramount importance and constitute major outcome measures in research studies. However, to date, these patellar tendon properties have not been described in sheep, and there is therefore a need to document them in a population of research animals.

The aim of this study was therefore to document the properties of the healthy patellar tendon in research sheep, assessing eventual changes with age and weight; the result was sought in terms of stiffness, addressing in this way the whole tendon system and not only the material it is composed by.

## Material and methods

### Animals

Thirty-four ewes (Ile de France; n = 34), from the Ovine Research Center of the University of Namur, were used. Their age ranged from 2 to 10 years, and their weight from 65 to 95 kg. The experimental protocol 10150MU was approved by the local ethical committee for animal welfare. All animals had no history of hindlimb lameness. They were assessed by palpation and observation. No swelling or pain of the stifle was observed. Animals were not lame before euthanasia.

Animal were sorted into three categories of age (1–2yo, 3–5yo, 6–10yo).

### Gross anatomy

Animals were sacrificed by intravenous administration of pentobarbital (150 mg/kg). Both hind limbs were transected at the level of the mid-femur within 1 hour of death. Soft tissue, including skin and muscles were removed. The patellar tendon and its attachments on the patella and the tibial tuberosity were carefully dissected and observed to confirm the absence of lesions or abnormalities.

The joint capsule, collateral ligaments and cruciate ligaments were transected. The knee joint was disarticulated, and the tibial tuberosity and patella were transected at 1.5 cm from their respective tendon attachments. Bone attachments were preserved so as not to affect the tendon insertion areas that are fundamental for mechanical load-transfer.

Each harvested specimen consisting of an intact ‘patella-patellar tendon-tibial tuberosity’ unit was individually wrapped in moistened gauze (with 0.9 % w/v NaCl), sealed in plastic bags, individually identified by a number and stored at −20°C.

Four-mm thick osteochondral slabs in a coronal plane were obtained (medial femoral condyle, lateral femoral condyle, medial tibial condyle, lateral tibial condyle), before being processed for histology.

### Histopathology of cartilage

Histology is used as the gold standard to assess the articular cartilage of a joint, joint deterioration and joint ageing (Little et al. 2010; Vandeweerd, Kirschvink et al. 2013). Articular cartilage changes are associated with ageing and diseases such as osteoarthritis. In the current study, histology was performed to assess joint deterioration and confirm the sample was representative of a normal ageing population of sheep by comparison to results of previously published studies (Vandeweerd, Hontoir et al. 2013).

The osteochondral slabs were fixed in a 10% w/v neutral buffered formalin for 48 hours. The specimen were decalcified in 10% w/v formic acid/ 5% w/v formalin during 8 to 10 days, depending on softening of the slabs. After paraffin embedding, 7 μm sections were cut and mounted on Superfrost Ultraplus® slides that improve cartilage adhesion. The sections were thoroughly deparaffinized in several xylene washes and graded alcohols to 70% w/v ethanol, then stained in 0.04% w/v toluidine blue and counterstained in 0.1% w/v aqueous fast green FCF. Finally, the slides were dehydrated in two changes of 99% isopropyl alcohol and two changes of xylene before mounting in DPX (DPX mounting media for histology).

**Figure 1.**
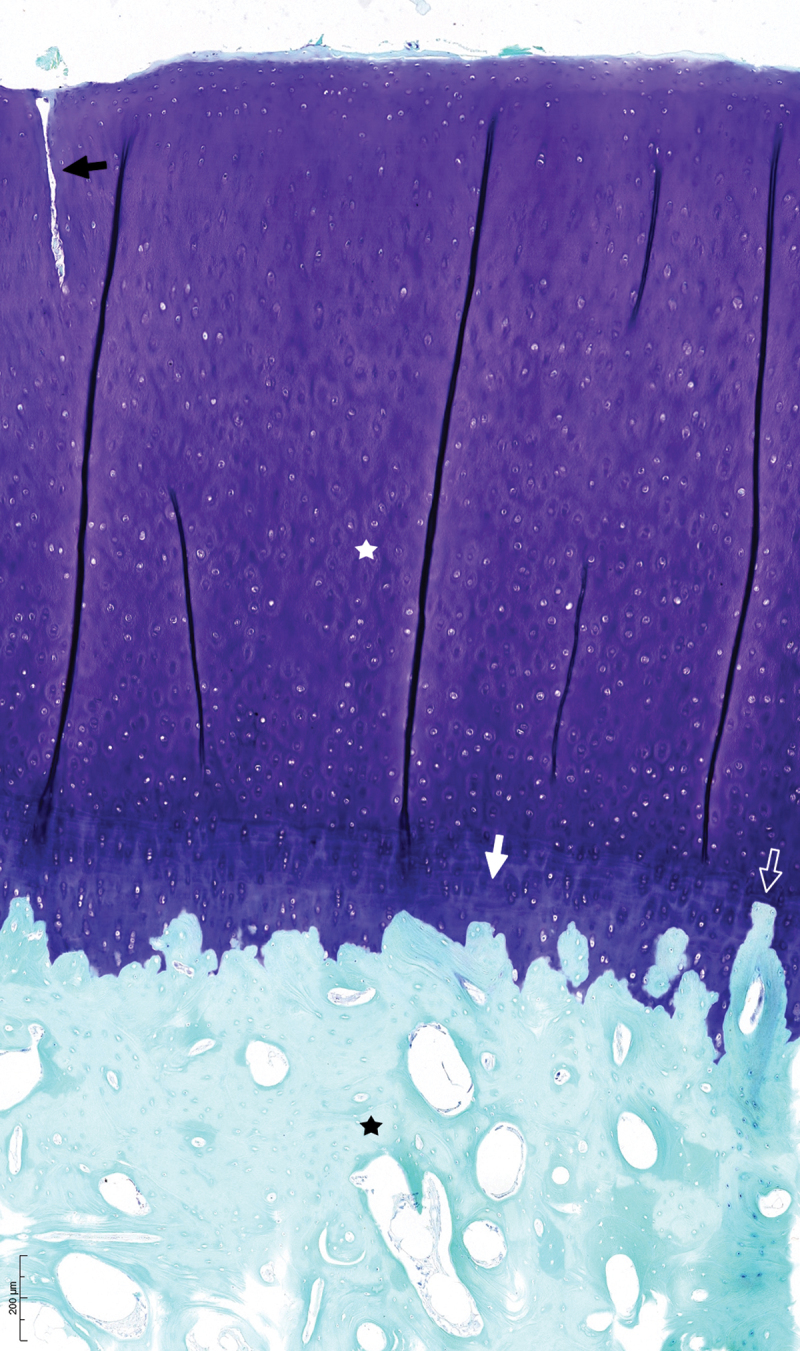
Toluidine blue/fast green stained section of cartilage scored as follows: Structure 4 (fissures to transitional zone 1/3 depth (black arrow)), Chondrocyte density 0, Cell cloning 0, Interterritorial toluidine blue 0, Tidemark 2 (duplicated tidemark (white arrow) with blood vessel penetrating through the subchondral bone plate (white open arrow)), with a total score of 6. Cartilage (white star). Subchondral bone (black star). Scale in lower left corner.

The OARSI recommendations were used for histological scoring of the cartilage (Little et al. 2010). Histological abnormalities included: structural defects (0–10), chondrocyte density (0–4), cell cloning (0–4), interterritorial Toluidine blue (0–4), tidemark (0–3), extent of the defect (0–5) with a total scoring of 0–30 (Figure 1). The histological grade per limb was determined by the summation of the total scores of the 4 anatomical regions in that limb.

### Biomechanical tests

**Figure 2.**
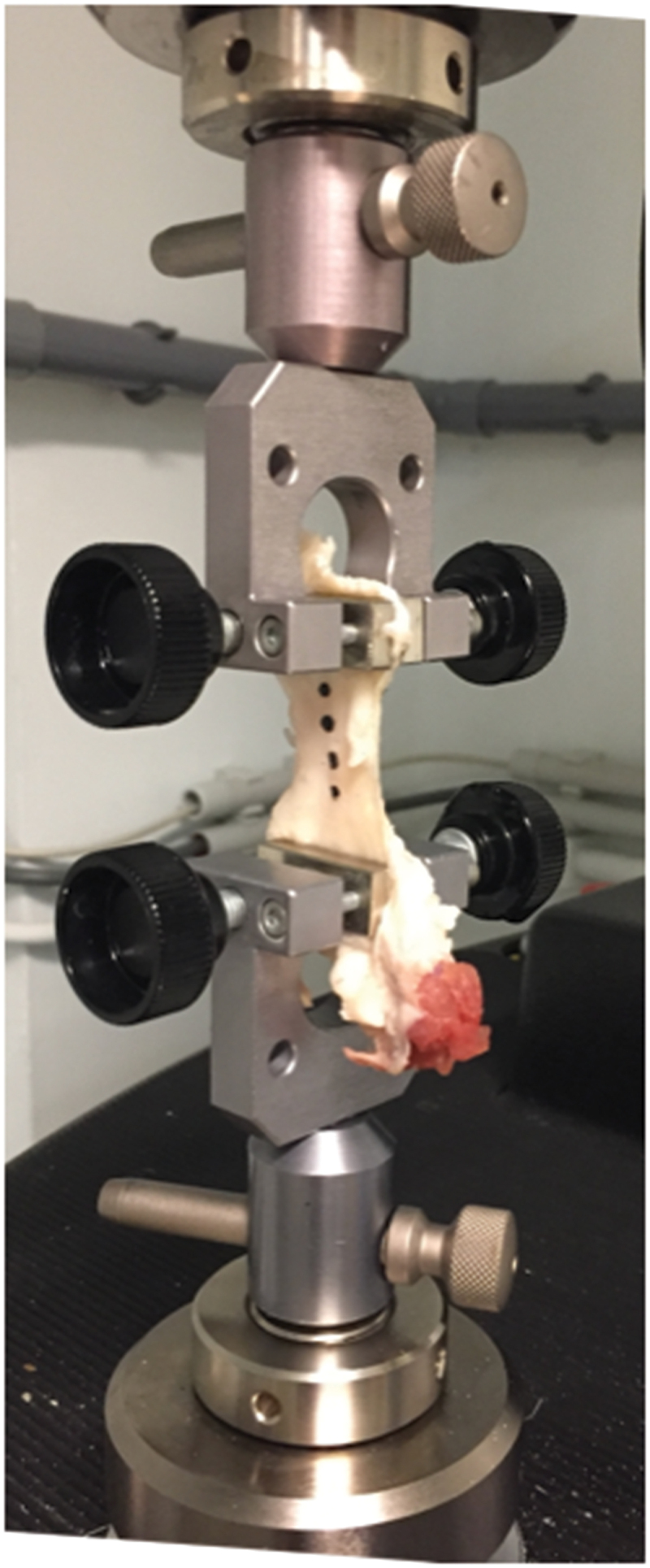
Patella-patellar tendon-tibial tuberosity unit fixed by two clamps. Four black ink dot markers were directly drawn onto the tendon specimen.

In thirty-four matched pairs of limbs (n = 68), a uni-axial tensile test was performed on patellar tendons following an in-house validated procedure (Innocenti et al. 2018). For all investigations, frozen ‘patella-patellar tendon-tibial tuberosity’ units were thawed to room temperature, cleaned thoroughly with 0.9 % w/v of NaCl and placed on a metal frame. The osseous parts of the specimen were cut with pliers to fit the clamp size. Four black ink dot markers were directly drawn onto each specimen in order to measure the displacement of the tendon tissue during the tensile test (Figure 2).

The camera was set to 100 fps. Force outputs were obtained via a dedicated 1 kN load cell (Lloyd Instruments Ltd). As the tendons had been manipulated and then stored at −20° C before the biomechanical tests, a series of 10 pre-conditioning stress-relaxation procedures was performed to ensure fibers alignment before the tensile testing. As recommended in the ASTM D638 standard (ASTM International 2003), a crosshead speed of 5 mm/min was applied for the tensile test. The axial force was recorded and paired with the relative displacement during the tests. The tensile test was performed until failure and the resulting force-displacement curve was analyzed: after the initial toe-region, the linear region of the curve (corresponding to the elastic region, i.e. the elongation of the helical structure of collagen (Kirkendall and Garrett 1997)) was defined thanks to the dedicated software: a linear fitting process was used to determine the boundaries of the linear region and this latter was studied to obtain the relative stiffness of the tissue (obtained from the slope of the curve in the selected region).

### Statistical analysis

In order to examine the normality of data (patellar tendon stiffness and histological scores of articular cartilage), Kolmogorov-Smirnov and Shapiro-Wilk tests were applied. Non-parametric tests were used, due to the non-normality of the data. The Wilcoxon signed-rank test was used to compare observations that were not independent (difference in patellar tendon stiffness between left and right hindlimbs). One limb was randomly chosen within each pair and data obtained from that limb (tendon stiffness, articular cartilage histological grades) were used for analysis. Correlations between patellar tendon stiffness and other variables (age, weight, histological grades) were assessed by Spearman correlation coefficient. Data were collected in Microsoft Excel and analysed using Graph Pad Prism 8. A p-value below 0.05 was considered to indicate a statistically significant difference.

## Results

### Cartilage histopathology

Among the 272 (4x68) histological slices, none presented artefacts or inadequate coloration that may have prevented correct interpretation. We found no significant difference in limb histological total scores between left and right limbs (p = 0.93).

The histological scores (median, minimum, maximum) were, however, different between regions of interest: medial tibial condyle (7, 2, 17), lateral tibial condyle (6, 2, 18), medial femoral condyle (6, 2, 21) and lateral femoral condyle (5.5, 1, 13). Among the total number of lesions, we found 36.2% in the medial femoral condyle, 6.8% in the lateral femoral condyle, 37.9% in the medial tibial condyle and 15.5% in the lateral tibial condyle. Among the histological abnormalities, beside the total score, structural defects and tidemark increased the most with age and weight.

There was a significant positive correlation between body weight and histological score (r = 0.51; p = 0.002), and between age and histological score (r = 0.67; p < 0.0001).

### Tendon stiffness

**Figure 3.**
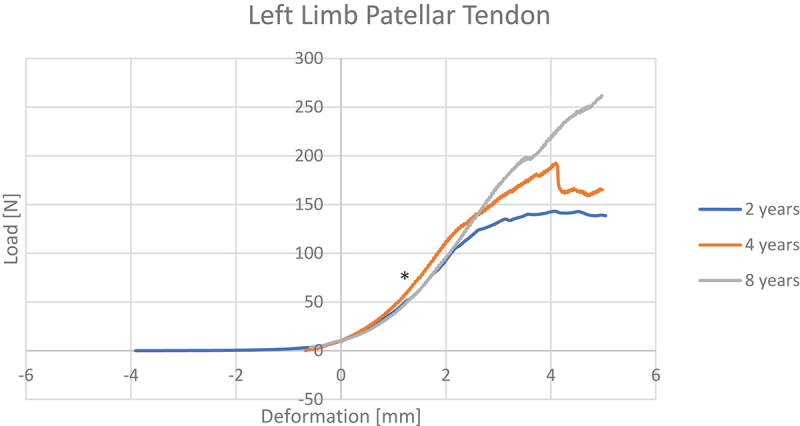
Stiffness curve of the left patellar tendons related to three different age groups: 2 yo (year old), 4 yo and 8 yo. * = linear region of the curve.

Our tests confirmed that the patellar tendon has the typical ‘region’ behavior, with a non-linear one (toe-region) followed by a linear region preceding the eventual damaging of the fibers and consequent failure of the tendon (Figure 3).

The mean stiffness value of the 68 tendons was 52.44 N/mm (SD 35.62, SD = standard deviation).

**Figure 4.**
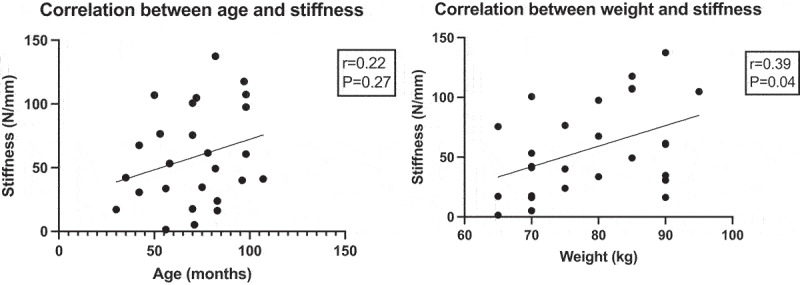
Correlation plot between age and stiffness (left) and between weight and stiffness (right). Correlation was positive but not significant between stiffness and age and statistically-significant positive between body weight and stiffness.

We found a positive but not significant correlation between stiffness and age (r = 0.22, p = 0.27) (Figure 4). We also found a statistically-significant positive correlation between body weight and tendon stiffness (r = 0.39, p = 0.04) and between articular cartilage histological scores and tendon stiffness (r = 0.47, p = 0.02)(Table 1).

**Table 1.** Weight, histological scores and stiffness related to three different age groups: A1 (1–2 yo (year old)), A2 (3–5 yo) and A3 (6–10 yo).

	A1(1–2yo)	A2(3–5yo)	A3(6–10yo)
Weight (kg, mean±SD)	74.47±8.8	77.79±9.23	77.63±8 .44
Histological score (median, minimum, maximum)	4(0,4)	9(4,47)	22.5(6,69)
Stiffness (N/mm, mean+/SD)	25.51±14.57	53.14±35.6	47.2±30.11

## Discussion

In research studies, the biomechanical characteristics of the tendon, like elasticity and stiffness, represent major outcome measures. Due to the biomechanical similarity of the ovine stifle joint and the human knee (Herfat et al. 2012), the patellar tendon of the sheep can be adopted as a large animal model for human tendon disease investigation.

In our study, as previously described in a population of normal sheep (Vandeweerd et al. 2013a), the histological changes of the stifle articular cartilage differed between anatomical regions of the joint, and the proportions of defects were around 30 % of lesions identified at the medial femoral condyle and 30 % at the medial tibial condyle. In addition, a significant positive correlation between age and histological grade was identified. This indicated that our sample could be considered representative of a normal ageing population of sheep. We also found a significant positive correlation between body weight and histological grade of cartilage. Overweight has previously been described in man as a risk factor for articular joint deterioration (Zhang and Jordan 2010; Vidal-Bralo et al. 2016; Valdes and Stocks 2018).

The mean value of stiffness was 52.44 N/mm (SD35.62) in our patellar tendons. Our results are quite similar to those found in a previous ex-vivo study (46–65-80 N/mm) performed in ovine patellar tendon (Kasperczyk et al. 1991).

Our study showed a positive but not significant correlation between age and tendon stiffness. A study on canine patellar tendons demonstrated progressive stiffening of connective tissues with ageing (Haut et al. 1992). A study performed in rats showed that normal ageing altered the passive biomechanical properties of the muscle-tendon unit in Achilles tendons, with a decreased relaxation response and increased stiffness in the tendons of middle-aged animals (Plate et al. 2013).

A study conducted in New Zealand White rabbits, however, showed no evidence of age-related decline in the biomechanics of healing tendons (Dressler et al. 2006).

The association between increased tendon stiffness and ageing may have different explanations. Tendons contain tenocytes that secrete matrix components such as elastin, proteoglycans and collagen (90% collagen type I). Several of these components may be involved in tendon stiffness changes with ageing.

Two different studies hypothesized that tensile stiffness changes in tissue are most likely due to changes in higher collagen structures rather than stiffness increasing at the fibril level (Li et al. 2013; Fessel et al. 2014). The difference in biomechanical properties between energy-storing tendons (i.e. the patellar tendon) and positional ones could also originate from a difference in the geometrical disposition of collagen within the fascicles (Shearer et al. 2017). The compliance of energy-storing tendons may be due to the helicoidal fibril disposition of their fascicles rather than differences in their fibril Young’s modulus or crimp angle. Energy-storing tendons present a less stiff interface between the tendon fascicles enabling a greater fascicle sliding. This may account eventually for increased failure strain (Thorpe et al. 2015). Concomitantly fascicle sliding has been described to decrease with ageing (Thorpe et al. 2013). Even if no decrease in collagen content was noted in ageing tendons, less organized collagen fibers containing an increased number of larger fibrils were observed (Gehwolf et al. 2016).

In mice, older tendons were stiffer than young tendons. It was suggested that the consequences of ageing on mechanical properties could be due to advanced glycation end products (AGEs). The level of AGEs was higher in aged mice compared to younger ones (Wood and Brooks 2016). Denaturation and crosslinking of collagen result from the formation of AGEs. These collagen crosslinks limit fiber-fiber and fibril-fibril sliding, reduce the viscoelasticity of the tendon and increase the tendon stiffness. This may explain the increase in tendon stiffness with ageing as observed in another study conducted in rats (Gautieri et al. 2017). Some in vitro studies (Reddy 2004) showed an association between AGEs and tendon mechanical properties. However, a clinical study conducted in man failed to associate tendon AGEs and in vivo patellar tendon mechanical properties (Eriksen et al. 2019).

Proteoglycans also are involved in viscoelastic changes in aged tendons. Decorin is the most abundant proteoglycan in the small leucine-rich proteoglycan family (SLRP) in tendons. Decorin regulates the assembly of collagen I which is the primary structural unit and transmits mechanical force (Xu et al. 2018). The absence of decorin leads to an abnormal collagen fibrillogenesis, decreased tendon strength and stiffness (Danielson et al. 1997). Decorin and biglycan are essential regulators of collagen fibril and matrix assembly and provide overlapping functions rather than single deficiency-related abnormalities. A study in a both decorin and biglycan gene expression knockout mouse model showed changes in structural properties as a shift to larger diameter fibrils with increased heterogeneity, and altered mechanical properties as decreased stiffness (Robinson et al. 2017). A study carried out on old rats found decreased proteoglycan 4 and elastin mRNA expression in tendons was responsible for the increased tendon stiffness observed with ageing through reduced gliding properties of fascicular sheets (Kostrominova and Brooks 2013).

An age-associated reduction in the functional fitness and metabolism of tendon stem cells may be partially responsible for an ageing induced deterioration of the structure, composition and mechanical properties of tendon. Some authors described ageing as an ‘anarchy of stem cells’, with a decrease in the number and the functional fitness of tissue-specific stem cells (Fukada et al. 2014).

A former study showed a higher stiffness in aged rat tendon-derived stem cells (TDSCs) than that of young TDSCs (Kiderlen et al. 2019).

In the current study, we observed a significant positive correlation between tendon stiffness and increasing weight in sheep. In man, higher tendon injury incidence (Kelly et al. 2001; Savarese et al. 2010) and lower patellar tendon stiffness (Tas et al. 2017) were reported in overweight and obese human individuals. Another study showed that patellar tendon stiffness was higher in males than in females, and obesity decreased patellar tendon stiffness in females contrary to males (Tas et al. 2018). A different study reported instead that higher BMI was likely to be associated with greater tendon stiffness particularly in young men (Tomlinson et al. 2021).

Despite the possible correlations, strict comparison between species is complicated because lipid metabolism seems to be involved in tendon stiffness and this factor is different between omnivores and ruminants. A positive association between increased adiposity and tendinopathies was shown in man (Gaida et al. 2009). A study conducted in mice observed an accumulation of lipid droplets in aged Achilles and tail tendons, with an increased expression of adipogenic markers and reduced expression of beta-catenin, the latter being a regulator of adipogenesis (Gehwolf et al. 2016).

Tendolipomatosis, may lead to tendolipomatosis-tendinopathy by lipid cell deposition in the tendon tissue (Jozsa et al. 1984; Kannus and Jozsa 1991). Tendolipomatosis was predominantly reported in the quadriceps or patellar tendons. The lipid cells deposit in the tendon among the collagen fibers and may disrupt the cohesion of the collagen framework, thus weakening the tendon and increasing the risk of its mechanical failure. Tendon-weakening is further exacerbated in overweight or obese individuals where the larger body mass increases the mechanical loading of the tendons.

Beside the effect of local lipid cells, the metabolic effects of increased adipose tissue might be responsible for lower tendon stiffness in man. The profile of adipokines (cytokines released by adipose tissue), and other cytokines expressed in obese individuals generally indicates a pro-inflammatory state (Battery and Maffulli 2011). Cytokines may originate from the infrapatellar fat pad (Ushiyama et al. 2003). There is a relation between cytokines released by infrapatellar fat pad (Hoffa’s fat pad) and knee osteoarthritis (Pottie et al. 2006). A larger infrapatellar fat pad (Culvenor et al. 2011) and systemic adiposity (Gaida et al. 2009) was associated with tendinopathy (Culvenor et al. 2011).

Recently, shear wave ultrasound elastography has been evaluated to assess the mechanical properties of muscles and tendons, including stiffness, based on the speed with which induced shear waves propagate in the tissue of interest. The effect of hyperlipidemia on the patellar tendon stiffness has been investigated by this method; and a moderate positive statistically significant correlation was found between patellar tendon shear wave velocity and low-density lipoprotein independently of body mass index (Torgutalp et al. 2020).

Those various and contradictory results indicate that different variables should be considered to assess the effect of weight in a species, such as the sheep, including biomarkers of lipid metabolism. This should be taken into account in future studies. One other limitation of the current study is that we measured the stiffness over the entire bone-tendon-bone unit whilst regional variations in stiffness may occur.

However, in the scope of an ovine model of patellar tendon, this study showed that the stiffness of the healthy ovine patellar tendon increases significantly with weight. It also increases, but not significantly, with age.

Even if the parameters taken into consideration were only a part of the huge amount to address when dealing with soft tissues, this study achieved promising results in terms of characterization of biomechanical properties of healthy tendons, providing useful reference values and establishing the basis for future biomechanical tests on healing tendons in sheep. The most appropriate sheep population for those future studies would be young, non-overweight adults presenting with no lameness. Non-invasive techniques such as shear wave ultrasound elastography warrant investigation.
